# Directed evolution of hydrocarbon-producing enzymes

**DOI:** 10.1186/s13068-025-02689-4

**Published:** 2025-08-12

**Authors:** Jochem R. Nielsen, Joseph Kennerley, Wei E. Huang

**Affiliations:** 1 Department of Engineering Science, University of Oxford, Oxford, OX1 3PJ UK; 2 Oxford SimCell Ltd, Begbroke Science Park, Begbroke, OX5 1PF UK

**Keywords:** Directed evolution, Hydrocarbons, Drop-in fuel, Enzyme engineering, Screening and selection, Growth-coupling

## Abstract

Enzymes capable of catalysing the production of hydrocarbons hold promise for sustainable fuel synthesis. However, the native activities of these enzymes are often insufficient for their exploitation in industrial bioprocesses. Enzyme engineering approaches including directed evolution (DE) can be used to improve the properties of enzymes to meet desirable standards for their industrial application. In this review, we summarise DE methods for engineering hydrocarbon-producing enzymes, including both screening- and selection procedures. The efficacy of DE depends on several factors, including sensitive and accurate detection of enzyme activity, the throughput of screening or selection steps, and the scale of diversity generation. Although DE is a well-established approach, its application in engineering hydrocarbon-producing enzymes has not been widely demonstrated. This can be attributed to the physiochemical properties of the target molecules, such as aliphatic hydrocarbons, which can be insoluble, gaseous, and chemically inert. Detection of these molecules in vivo presents several unique challenges, as does dynamically coupling their abundance to cell fitness. We conclude with a discussion on future directions and potential advancements in this field.

## Introduction

Earth’s visibly changing climate demands global attention and necessitates reduced greenhouse gas (GHG) emissions across diverse sectors. 20–25% of GHG emissions originate from the unsustainable combustion of hydrocarbon fuels in personal and commercial transport sectors [21]. While electric vehicles significantly reduce CO_2_ emission and contribute to sustainable development, hydrocarbon fuels produced from carbon neutral processes still hold an advantage due to their high energy density. For example, hydrocarbon fuels remain the primary energy source for the aviation sector [20]. Over the past decades, transportation fuels in some economies have been replaced to some degree with bioethanol and biodiesel; for example, Brazil, where biofuels represent approximately 25% of transport fuel consumption [22]. Although these positively contribute to reducing net emissions, the chemical composition of these fuels is not identical to fossil hydrocarbons, necessitating technical developments in engine technology, such as flex-fuel engines, to facilitate widespread adoption. Otherwise, such fuels must be blended with conventional fuels before use in most internal combustion engines (ICEs), reducing their scalability and resulting in the so-called “blend wall”. In addition, concerns about arable land requirements for biofuel production limit their attractiveness and scalability as fossil fuel alternatives.

The biological production of aliphatic hydrocarbons presents a promising solution to some of the longstanding issues in the biofuel sector. Firstly, the production of chemically identical hydrocarbons (‘drop-in’ fuels) alleviates the blend wall issue, allowing either neat usage or significantly increased blend percentages. Secondly, microbial cell factories can, either natively or through genetic engineering, consume low-cost substrates including various waste streams, CO_2_, or substrates derived from CO_2_ such as acetate and formate. This can shift bio-based processes from the current mostly crop-derived substrates towards truly sustainable production [78], alleviating our reliance on a finite lithospheric carbon reservoir.

Natural microbial bioproduction of accessible hydrocarbons at industrially relevant titres, rates, and yields (TRY) is rare [40]. Further, most widely studied microbes produce no immediately useful fuel molecules, including gasses such as butane and propane (main components in liquified petroleum gas, LPG) or liquid C_8_–C_16_ n-alkanes (components of petrol and kerosene fuels) [110, 111]. This necessitates metabolic engineering to establish a biosynthesis pathway for the desired product, which ideally will require minimal subsequent processing for compatibility with existing fuel blends. Some product formation can often be achieved this way, but the resulting TRY metrics are usually far below those required for commercial exploitation. Fortunately, the modern synthetic biologist has a variety of tools available to them for targeted alteration of cell metabolism, enabling the application of engineering principles to cells to improve production. Although some standard techniques can be used to improve TRY by redirecting carbon flux towards product and balancing cofactor availability, sometimes the (terminal) enzymes involved directly in product synthesis remain limiting [4, 57].

In these cases, enzyme engineering approaches can be used to alter enzyme solubility, activity, (thermo)stability, or substrate specificity, potentially leading to improvements in production [65, 67]. Strategies including rational and semi-rational design can be used to target and alter specific regions or residues to achieve a desired change to an enzyme’s properties. Rational design benefits from a comprehensive understanding of the enzyme’s structure and catalytic mechanism, traditionally achieved by determining a crystal structure of the enzyme in question [45], [102]. A wide variety of enzyme classes have been successfully engineered using small rationally designed ‘smart’ libraries using structural information as input [14]. However, even with structural information success is not assured, rational enzyme engineering can lead to disappointing results when designed alterations have unexpected effects, such as a radical loss in stability or activity due to unforeseen interactions between residues. Besides this, the determination of an enzyme’s crystal structure is laborious, expensive and, in some cases, cannot be realised at all.

The development of in silico protein structure prediction and protein design tools have vastly reduced our reliance on experimental protein structure determination, often representing the bottleneck to this approach. Considerable advancements in this field include the development of Rosetta- and, more recently, AlphaFold algorithms [39, 72]. AlphaFold, a machine-learning based protein structure prediction model, is capable of achieving unprecedented speeds and accuracies in predicting protein folding, enabling researchers to predict structures for almost all known proteins [89]. Although the volume of structural data generated by AlphaFold has become vast, gathering information concerning the relationship between a predicted enzyme structure and its biological performance—referred to as labelled data—remains a challenge and hinders machine-learning approaches [90, 98]. To address this challenge, advances are also being made in the prediction and improvement of protein functionality using unlabelled data [51]. In most cases however, to truly elucidate this relationship either a function must be experimentally validated and assigned to an enzyme structure, or a desired functionality must be identified and isolated from a pool of diverse enzyme structures, the latter of which is achieved through screening or selection.

Semi-rational enzyme engineering methods permit researchers a more naïve starting point in the engineering pipeline compared to full rational design. These techniques often rely on sequence-level data (as opposed to structural data) to help select residues to mutagenise, increasing the chance that a mutation has a functional effect and drastically reducing library sizes compared to methods using random mutagenesis. For example, phylogenetic datasets analysed through multiple sequence alignments (MSAs) can be interrogated to identify evolutionary ‘hotspots’ within sequences, uncover co-evolving residues, and generate ancestral protein sequences, the latter of which often display higher thermostability and altered substrate specificities [32, 61]. Combinations of (predicted) structural information and evolutionary sequence-level have also been successfully used to engineer a variety of enzyme properties [87].

An alternative approach that can function without any prior knowledge of an enzyme’s structure or catalytic mechanism, and which can interrogate a much wider solution space for high-performing mutants, is random mutagenesis combined with directed evolution (DE), which uses iterative rounds of mutagenesis and screening or selection to identify enzymes with desirable traits from a large pool of variants [5]. To use this approach, the desired activity of the enzyme must be measurable (screenable), allowing high-performing variants to be screened and isolated. Alternatively, the target activity must contribute to fitness (selectable) within a population of cells carrying enzyme variants, enabling those cells with desirable variants to become the population’s dominant genotype [66]. This requires the development of a robust method to screen or select for desirable enzymatic activity, which in itself is the most challenging aspect of DE.

Screening and selection methods designed for DE can be used to identify desirable variants from the relatively small libraries often generated by (semi)-rational enzyme engineering methods. However, when random mutagenesis is used for diversity generation, then not only must the DE method be robust, but it must also allow for a high throughput. Random mutations have a vastly lower chance of being beneficial as opposed to neutral or detrimental, concomitantly requiring a vastly higher throughput to identify beneficial mutations in a population of variants [12]. While high throughput is required for any DE approach utilising random mutagenesis, establishing a DE method for hydrocarbon production faces some unique challenges compared to other classes of enzyme products, which we discuss in this review.

The application of directed evolution methods to engineer biocatalysts has been well described elsewhere [6, 13, 71, 105], as have advancements specifically in the field of in vivo directed evolution [26, 68, 82, 92]. In this review, we summarise the development and use of DE approaches specifically for hydrocarbon-producing pathways and enzymes. Finally, we provide perspectives and suggestions for the further development of the field, hopefully accelerating the use of DE methods to establish commercially viable sustainable fuel production.

## Hydrocarbon biosynthesis

**Fig. 1:**
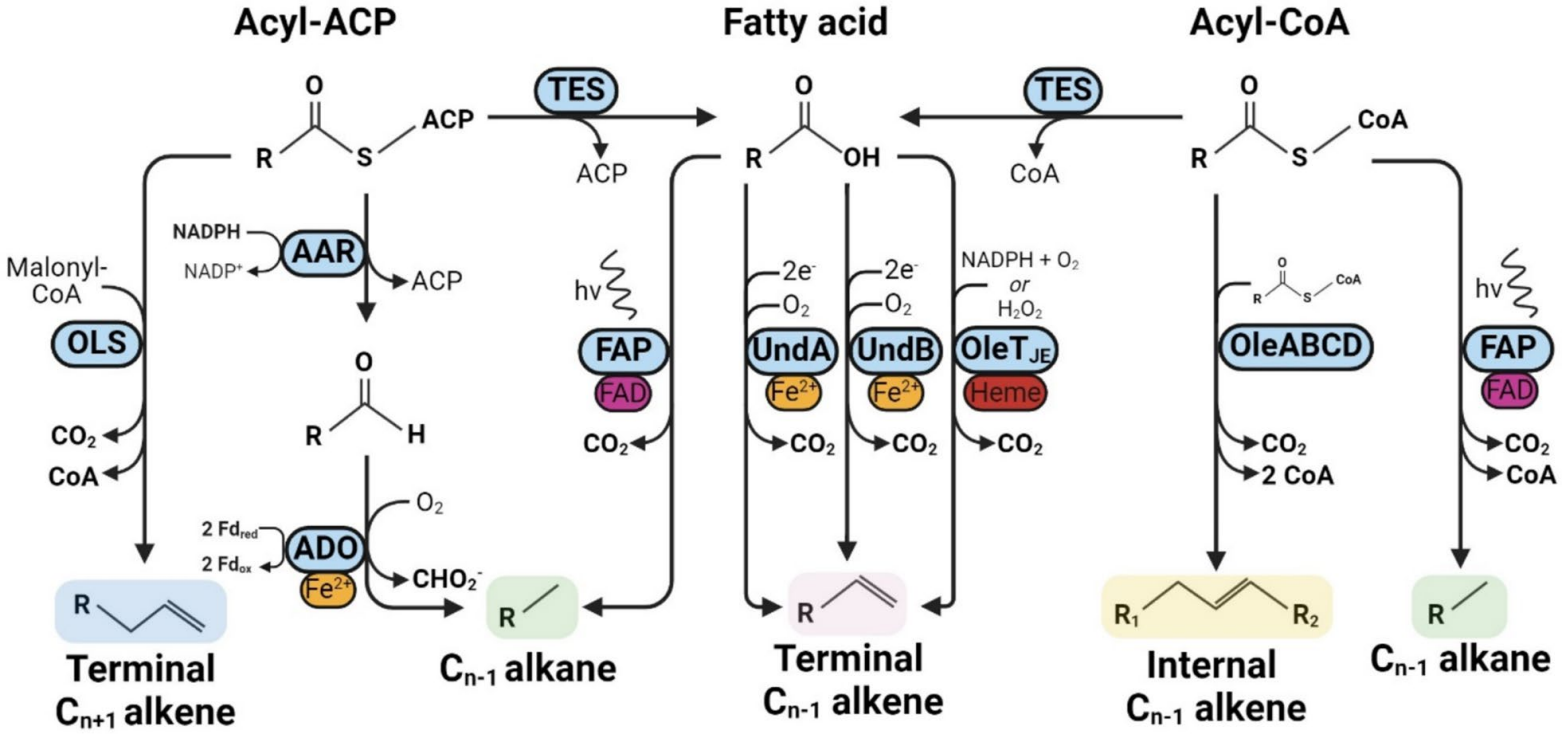
Enzymatic pathways for hydrocarbon biosynthesis. All currently known biosynthetic pathways for alkene and alkane biosynthesis, abbreviations and native substrate specificities in terms of carbon chain length are indicated in parenthesis where relevant. Thioesterase (TES), olefin synthase (OLS, C_20_), acyl-ACP reductase (AAR), aldehyde deformylating oxygenase (ADO, C_4–_C_18_), fatty acid photodecarboxylase (FAP, C_12_–C_18_), α-olefin synthases UndA (C_10_–C_14_)/UndB (C_6_–C_17_)/OleT_JE_ (C_4_–C_20_) and head-to-head fatty acid condensation enzymes OleABCD (C_25_–C_32_). Cofactors, coenzymes, prosthetic groups or metal ions required for catalysis are indicated including nicotinamide adenine dinucleotide phosphate (NADP^+^/NADPH), acyl carrier protein (ACP), coenzyme A (CoA), heme, Fe^2+^ and FAD (flavin adenine dinucleotide)

Various hydrocarbon-producing enzymes have been identified in nature. Already since the 1960’s waxes, alkanes and alkenes have been detected in insect, plant and cyanobacterial extracts. These hydrocarbons have disparate functions, ranging from protection against desiccation in insects and plants to promoting cell wall flexibility and salt tolerance in cyanobacteria [10, 35, 37, 46, 106]. Research efforts over the last two decades have led to the initial identification, characterisation, and application of several of the metabolic pathways responsible for hydrocarbon biosynthesis (Fig. 1). For the purposes of this review, we focus on bacterial hydrocarbon synthesis enzymes since many of these have been heterologously expressed and their catalytic activity characterised. In addition, we use the term ‘hydrocarbons’ to refer to aliphatic hydrocarbons (alkanes/alkenes), since these compounds are abundant in natural gas, gasoline, kerosene, and diesel, hence they have significant potential as drop-in fuels.

The constituent molecules of a drop-in compatible biofuel should be chemically similar to those found in a petroleum counterpart; primarily paraffins (n-alkanes, generally with carbon length C_4_–C_12_ for gasoline, C_9_–C_25_ for diesel, and C_5_–C_15_ for jet fuel), olefins (n-alkenes), isoparaffins (branched alkanes), naphthenes (cycloalkanes), and aromatics (mono- or polycyclic unsaturated hydrocarbons). n-Alkanes are a major component of transportation fuels and biologically derived alkanes can directly displace fossil-derived alkanes without impacting fuel quality or requiring changes to engine infrastructure, hence why biosynthetic pathways for producing n-alkanes are attractive candidates for optimisation and upscaling.

Aromatic fuel hydrocarbons—such as benzene, toluene, and xylene (BTX)—cannot yet be reliably produced directly using microbes, though biosynthesis of toluene has been observed and some key metabolic enzymes identified [9]. Derivatives of these aromatics, including phenol [96] and vanillin [63], have been biosynthesised, and directed evolution has been applied to biosensors for some derivatives [28], but there have been no significant attempts so far to use directed evolution to improve biological production of aromatic fuel hydrocarbons.

### Terminal alkene biosynthesis

The first isolated enzyme responsible for terminal alkene biosynthesis was found to be a cytochrome P450 from the bacterium *Jeotgalicoccus* sp., dubbed OleT_JE_, which catalyses the decarboxylation of fatty acids, producing alkenes [74]. Its substrate specificity was found to be extremely broad, enabling the production of short-, medium- and long chain C_n-1_ olefins (C_4_–C_20_) from the respective fatty acids with concomitant release of CO_2_ [24]. The OleT_JE_ enzyme also displayed flexibility in terms of its electron source, which can either be H_2_O_2_ without the need for oxygen or via a reduced electron carrier in the presence of oxygen [54].

Another terminal alkene biosynthetic pathway was discovered in *Synechococcus* sp. PCC7002, which was initially observed to produce C_19_ alkenes through an unknown mechanism [97]. Elucidation and characterisation of the pathway revealed that a multidomain protein named olefin synthase (*ols*), with homology to type I polyketide synthases, was responsible for C_19_ terminal alkene synthesis through an elongation and subsequent decarboxylation mechanism [59]. This cyanobacterium did not accumulate free fatty acids over C_18_ in length, which excluded the possibility that direct decarboxylation of free fatty acids was responsible for C_19_ alkene biosynthesis. Instead, the synthesis of C_19_ alkenes was found to occur through additional acyltransferase activity which elongated a C_18_ fatty acid with malonyl-CoA, followed by a final decarboxylation step yielding a C_19_ product.

A reverse genetics approach was used to identify the enzymes responsible for 1-undecene biosynthesis in *Pseudomonas*. This revealed a mononuclear iron oxidase, UndA, which utilised O_2_ and a reducing agent to decarboxylate fatty acids to corresponding terminal alkenes [76]. Further study later revealed it to constitute a new class of diiron decarboxylase [56]. The UndA enzyme could convert C_10_–C_14_ fatty acids to their corresponding C_n-1_ 1-alkenes but was unable to utilise substrates with chain lengths ≤ C_8_ and ≥ C_16_, indicating a narrow substrate specificity range [76]. Upon further study another 1-undecene producing enzyme was found by the same group, dubbed UndB, which also catalysed the oxidative decarboxylation of fatty acids. This enzyme was found to have a broader substrate specificity than UndA, ranging from C_6_–C_17_ fatty acids, although with a preference for C_10_–C_14_ [75]. In contrast to UndA, the UndB enzyme is membrane-bound, which has hindered biochemical analysis of its catalytic mode of action. However, it displays homology to other diiron desaturases, making it likely that UndB also contains a diiron core. The biosynthesis of 1-alkenes in yeast was achieved using UndB [110, 111], and it was found capable of catalysing decarboxylation of dicarboxylic acids [99].

Compared to OleT_JE_ and UndA, in vivo expression of the membrane-bound UndB enzyme results in higher production of 1-alkenes both in *E. coli* and *S. cerevisiae* [52]. Most engineering efforts have focussed on redox partner optimisation for the monooxygenase function of OleT_JE_ [24, 29], [54]. Only one recently published pre-print describes the use of DE to evolve UndB [94]. The authors described using in silico guided residue selection and alanine scanning to identify UndB variants with improved substrate specificity towards C_9_–C_14_ dicarboxylic acids, achieving an approximate fivefold improvement in conversion of C_10_ dicarboxylic acid to 1,7-octadiene. Apart from this pre-print, no other work has reported using DE for the engineering of OleT_JE_, Ols, UndA or UndB.

### Internal alkene biosynthesis

The biosynthesis of internal alkenes occurs via the head-to-head condensation of fatty acids. The *oleABCD* genes from *Micrococcus luteus* were found to be responsible for biosynthesis of C_27:3_ and C_29:3_ alkenes, via consumption of two fatty acyl-CoA and the release of one CO_2_ [8]. Soon after, more *ole-*type operons were discovered in a variety of bacteria. Some of these were characterised, revealing that a wide array of olefin products can be generated through this pathway, ranging from C_24_–C_31_ in length and with up to 9 unsaturated bonds [86].

### Alkane biosynthesis

Saturated alkanes are biosynthesised in plants—primarily as very-long-chain (VLC) alkanes which comprise cuticular waxes—most notably by ECERIFERUM 1 and 3 (CER1/CER3) in *Arabidopsis thaliana* [1, 11, 15]. In insects, an NADPH-dependent cytochrome P450 from the CYP4G family was shown to be a highly conserved enzyme involved in alkane biosynthesis [104]. Such enzymes, native to eukaryotes, are commonly expressed in yeast for functional characterisation [11], though specific applications for biofuel production have not been extensively explored. Eukaryotic enzymes can be difficult to express in prokaryotes, limiting their practical use in model bacteria like *E. coli*, though CER1 has been expressed in *Acinetobacter baylyi* as part of a two-stage process to convert CO_2_ to acetate and then acetate to alkanes [48]. The carbon chain length specificity of plant and insect alkane biosynthesis enzymes (C_20_–C_34_) exceeds the range required for drop-in kerosene- or petroleum fuels, although some products may be suitable as diesel replacements. Attempts to use these enzymes for scalable bio-based production of these long-chain alkanes will have to contend with limited substrate solubility. The vast majority of research on alkane biosynthesis circumvents this challenge by focussing on using and engineering cyanobacterial alkane biosynthesis enzymes.

In 2010, a landmark study by Schirmer et al. reported the identification and characterisation of enzymes responsible for alkane biosynthesis in the cyanobacterium *Synechococcus elongatus* PCC7942 [77]. The proposed pathway consisted of an Acyl-ACP Reductase (AAR) and a terminal enzyme initially designated an aldehyde decarbonylase, owing to its supposed side-product being carbon monoxide. This was corrected in 2011, when the side-product of its reaction was found to be formate rather than CO, resulting in a renaming of the enzyme to Aldehyde Deformylating Oxygenase (ADO) [50, 95]. The AAR enzyme produces free fatty aldehydes using NADPH as an electron donor and fatty acyl-ACP as a preferred substrate, although it can also utilise acyl-CoA. The resulting fatty aldehyde product is then deformylated by ADO to produce a C_n-1_ alkane and formate as a by-product. The terminal ADO enzyme requires input of four electrons to perform the deformylation reaction, which are preferentially delivered by a ferredoxin (Fd)/ferredoxin reductase (FNR) system [109]. An FNR/Fd pair is therefore hypothesised to be the endogenous reducing system for ADO in cyanobacteria. Native ADO displays a wide substrate specificity ranging from C_4_–C_18_ aldehydes, and several studies have successfully improved the production of short-chain alkanes through rational enzyme- and/or metabolic engineering [3, 19, 41, 43, 81]. For example, structure-based rational engineering of ADO lead to an approximate doubling of propane titre [43]. Besides chain length specificity, other engineering attempts have yielded ADO variants with a 2- to 4-fold increased *k*_*cat*_/*K*_*M*_ [93] and increased thermostability [80]. Having been a target of metabolic- and enzyme engineers for over a decade, maximum titres of the heterologously expressed AAR/ADO pathway have currently reached the g/L level in *E. coli* [16, 31]. However, production rates and yields still remain commercially infeasible: an earlier techno-economic study reported that biobased alkane production must achieve a theoretical yield of at least 90% to compare with ethanol in terms of cost efficiency [17]. Since most studies reported ADO to be significantly rate limiting it has become an attractive enzyme engineering target. Even so, only a handful of studies have used DE approaches to engineer ADO, which are described in later sections.

A more recently discovered alkane- and alkene-forming enzyme catalyses the light-driven decarboxylation of fatty acids or fatty acyl-CoAs [49, 83, 84]. This activity was exhibited by the *Chlorella variabilis* fatty acid photodecarboxylase (*cv*FAP), which displays a substrate specificity of C_12_–C_18_ fatty acids with a preference for C_16_ and C_17_ and requires blue light (~ 400–500 nm) for its activity. Rational engineering of the enzyme resulted in improved specificity towards short-chain product formation between 29 and 552-fold, allowing the light-driven generation of alkanes as short as ethane [103]. Only recently has the *cv*FAP enzyme first been engineered via DE, resulting in a comprehensive study where the enzyme was repurposed to enable highly selective decarboxylative radical cyclisation, achieving a 184-fold improvement in yield of cyclised product [38]. Despite the novel screening pipeline devised and used successfully in this work, product identification and quantification were performed via analytical techniques such as GC, GC–MS or HPLC. Consequently, the throughput of such an approach is still constrained and necessitates relatively small variant library sizes.

Although DE has not been widely used for engineering alkane biosynthesis enzymes, other work has instead focussed on alkane degradation. For example, iterative rounds of DE and screening were successfully used to engineer a P450_BM3_ enzyme towards a high specificity for propane monooxygenase activity, allowing the selective conversion of this gaseous n-alkane. This successful enzyme engineering campaign demonstrated that DE is a viable strategy for targeting the properties of alkane-interacting enzymes [30].

### Challenges facing directed evolution of hydrocarbon-producing enzymes

Establishing a DE protocol varies in difficulty, depending mostly on the physical and chemical properties of the target product, and the extent to which the target enzyme has been characterised in terms of its activity and expression in the host cell. The detection and accurate quantification of a target product for DE is subject to several requirements (Table 1). Hydrocarbons satisfy few or none of these requirements, depending on their specific chemical composition. The most notable difficulty originates from the hydrophobicity of the substrates and/or products, and their ensuing poor solubility in physiological conditions. For example, if a certain hydrocarbon detection system relies on the compound being in solution, a poorly soluble product may not be detected, resulting in a false negative reading. Similarly, if the hydrocarbon product is gaseous, it may simply evaporate and no longer be available for detection in solution. A core requirement for directed evolution is the strong linkage between a detectable phenotype (e.g. enzyme-catalysed product formation) and the genotype of the responsible variant (its isolated sequence). The physiochemical properties of hydrocarbons can disrupt this linkage through evaporation, aggregation, and diffusion in and out of cells/vesicles. Hence, establishing a robust DE protocol for hydrocarbon biosynthesis is particularly challenging.

**Table 1:** Properties of hydrocarbons in the context of directed evolution

Property	Ideal enzyme product for DE*	Hydrocarbons
Solubility	**High** Product is ideally highly soluble to enable interactions with proteins and/or other chemicals	**Low** Often insoluble in aqueous environments, can be gaseous or solid depending on chain length, tends to localise to the cell membrane or aggregate due to hydrophobicity
Compartmentalisation	**High** No extracellular leakage of the product. In case of cell lysis, released product cannot be taken up by living cells	**Low** Short chain hydrocarbons are volatile, medium- and long chain hydrocarbons can passively diffuse through cell membranes. Can result in poor linkage between enzyme performance and sequence
Interaction between product and host metabolism	**Depending** For screening purposes, interaction with host metabolism should be low. For selection, coupling product formation to host metabolism can be a useful strategy	**Low** For most model organisms used as screening or selection platforms, hydrocarbons are not a native metabolite
Toxicity	**Low** Ideally, the target product should not be toxic	**Medium** Short and medium-chain hydrocarbons can be toxic. Longer chain hydrocarbons exhibit low toxicity

^*^Assuming a DE system is used where intracellular enzyme product concentration correlates with product detection response (screening) or growth (rate) (selection)

With these challenges in mind, attempts have been made to establish screening and selection protocols for hydrocarbon synthesis. The majority of these focus on detection and reporting of hydrocarbon production through a biosensor regulating expression of a reporter gene, although approaches involving selection instead of screening are starting to be adopted. We summarise the development of both these approaches for the DE of hydrocarbon biosynthesis enzymes in the following sections.

## Hydrocarbon screening methods

### Chemical screening methods

**Fig. 2:**
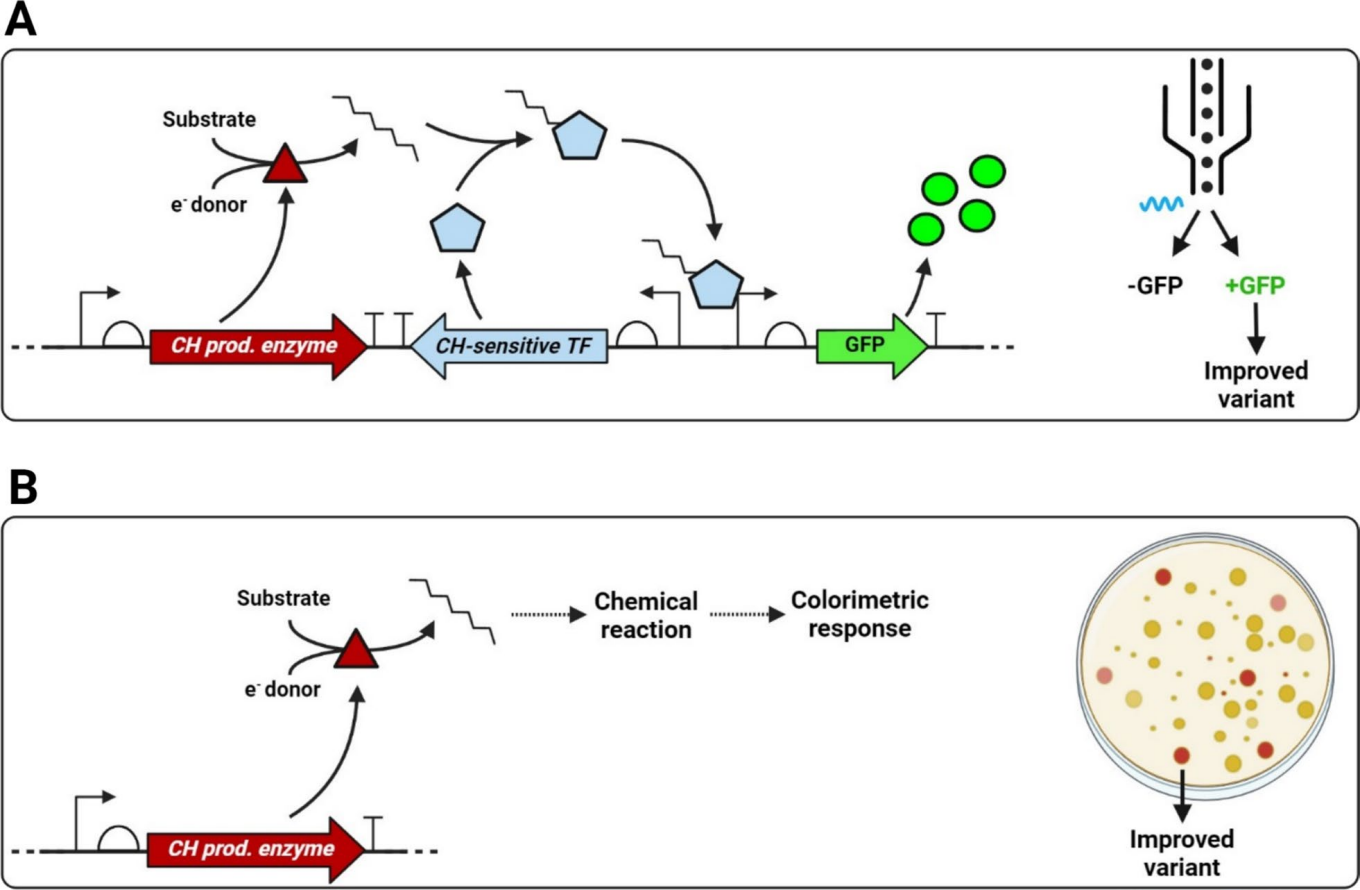
Screening methods for hydrocarbon biosynthesis enzymes. **A** Schematic representation of a synthetic genetic construct consisting of a hydrocarbon production gene (CH prod. enzyme), hydrocarbon-detecting transcription factor (CH-sensitive TF) and a TF-responsive element, in this case a GFP reporter gene. The expression of GFP is linked to hydrocarbon presence, and GFP-expressing cells can be sorted by a FACS procedure resulting in enrichment for improved hydrocarbon biosynthesis enzymes. **B** A chemical reaction with either the product (hydrocarbon) or substrate(s) can be coupled to a colorimetric response to identify isolates with (improved) activity

The use of chemicals that result in a colorimetric or fluorometric change upon activity of a target enzyme represents a straightforward and intuitive screening method for directed evolution (Fig. 2B). One such approach was used in the DE of a P450 decarboxylase of the CYP152 family (CYP-Sm46Δ29), where OleT_JE_ was included as a reference enzyme, using a colorimetric screen for H_2_O_2_ consumption [101]. The OleT_JE_ enzyme can use H_2_O_2_ as a sole electron and oxygen source to catalyse fatty acid decarboxylation, and the authors exploited this principle by expressing CYP-Sm46Δ29 and OleT_JE_ mutant libraries in catalase-deficient *E. coli* to identify H_2_O_2_-consuming variants. This approach revealed a CYP-Sm46Δ29 variant with a 3.2-fold improved k_cat_/K_M_ for lauric acid decarboxylation, although engineering of the OleT_JE_ enzyme itself did not yield any significant improvements.

### Transcription factor-based reporter systems

Many DE approaches are based on measuring the abundance of a product of interest via a reporter system. By pinpointing genes that play a role in either catabolism or anabolism of the desired product, genetic elements involved in transcriptional responses to its presence can be identified and characterised. These genetic elements can then become part of a toolkit used in synthetic constructs to drive reporter gene expression [107, 108]. For example, such a construct could consist of a hydrocarbon-sensitive transcription factor (TF) regulating the expression of a green fluorescent protein (GFP) (Fig. 2A).

Hydrocarbon-degrading microbes have been an abundant source of useful hydrocarbon-sensitive TFs with various affinities for a wide range of carbon chain lengths. For example, *Pseudomonas oleovorans* carries the alkS/P_alkB_ system and *Acinetobacter baylyi* carries the alkR/P_alkM_ system [69, 88], [107, 108]. Both systems exhibit comparable control architecture—activating expression of a native alkane monooxygenase catalysing degradation of environmental alkanes—and have been extensively characterised and repurposed for hydrocarbon detection. These systems have been used to detect hydrocarbon contamination in water using fluorescent or luminescent outputs [85], [107, 108], and to study cellular uptake of alkanes [33]. As pathways for biosynthesis of hydrocarbons are characterised and find use in bioproduction, so too have natural hydrocarbon biosensors been engineered to improve their sensitivity and specificity, in pursuit of improved hydrocarbon quantification in vivo (see Table 2).

**Table 2:** Transcription factor-based reporter systems for aliphatic hydrocarbon detection

Detector—reporter	Specificity	Dynamic range	Study findings	Reference
P_alkS_/alkR─P_alkM_-GFP	C_13_ – C_17_	1–3 mg alkanes per g CDW	Improved sensor performance, combined two alkane response elements	[100]
P_alkB_/alkS*─P_alkS_-eGFP	C_10_ – C_17_	50–500 mg/L	Engineered AlkS for improved response range, DE of AAR and ADO	[18]
P_L_λ/alkS*─P_alkS_-GFP	C_5_ – C_10_	Not indicated	Engineered AlkS for short-chain alkane sensitivity	[70]
P_alkRM_/alkR─P_alkM_-GFP *and* P-luxAB	C_12_ – C_18_	Detection of 40 mM alkanes, no range given	Monitoring aldehyde and alkane flux simultaneously	[47, 48]

A recent study reported the first example of alkane-sensitive biosensors being successfully used in DE, in this case to engineer ADO [18]. In this work, the alkane-responsive TF AlkS was engineered to increase its sensitivity to longer-chain alkanes. Four rounds of random mutagenesis were performed using bidirectional fluorescence activated cell sorting (FACS) screening, where cells displaying low fluorescence in the absence of alkanes were isolated first, followed by the addition of alkanes and isolation of the top 1% of fluorescent cells. After optimising the biosensor, the authors randomly mutagenised the AAR and ADO genes over four rounds and identified isolates displaying a 13-fold improvement in alkane titre. This work highlights the importance of optimising the hydrocarbon-detecting TF before attempting a DE approach, and the successful engineering of AAR-ADO reported in this study demonstrates the potential of biosensor-based DE strategies.

### Advantages and limitations of screening methods

A clear advantage of chemical screening systems is their simplicity. In addition, since they do not rely on biological parts for their functionality as is the case with TF-based screens, these methods are relatively robust, but also inflexible. A considerable disadvantage is their low throughput, as variants must usually be physically separated to discern differences in response. This limits many of these approaches to agar plate-based methods with a maximum throughput of ~ 10^3^–10^4^ variants per day, which is useful only for screening smaller libraries (e.g. smart libraries or site saturation mutagenesis libraries). In general, however, we expect that DE methods applied to randomly mutagenised libraries will move away from agar plate-based screening and towards higher-throughput methods.

An advantage of TF-based detection is that a response is generated based on direct interaction with the target molecule. It is also possible to swap out one biosensor system for another, for example if specificity towards a certain carbon chain length is preferred. Theoretically, hydrocarbon-responsive TFs can be engineered towards specificity for a wide range of chain lengths. It would therefore be possible to construct a library of TF variants with different specificities to a range of reaction products, enabling the swapping of detector modules to suit the needs of the DE campaign. Methods using TF-based detection could also be used to track the consumption of relevant substrates to infer biosynthesis rates of the product of interest. However, chain length specificity of the TF will be of similar importance as with product-detecting TFs, as discussed earlier. A TF-based system that detects the use of other molecules involved in the relevant reaction (such as electron donors) could also be envisaged, although interpretation of the sensor’s response may become complex due to the ubiquitous and cyclical nature of intracellular redox reactions. That being said, TF-based systems that are specific and sensitive independently of the substrate or product’s chain length would be highly useful and make them similar to chemical detection methods.

However, this approach also has several limitations. Firstly, although interaction with the target hydrocarbon is direct, any chosen TF will not elicit an equal transcriptional response to all hydrocarbons. The output of the DE campaign is therefore defined by the specificity and sensitivity of the chosen TF. To improve the chances of a successful outcome, TFs will likely need to be engineered for maximum affinity toward the desired hydrocarbon molecule prior to their use in DE studies, as exemplified in recent work [18]. In addition, as discussed earlier, most hydrocarbons can freely diffuse into and out of the cell. This presents an issue for hydrocarbon detection-based screening methods, especially when using longer cultivation times, as non-producing cells can take up exogenous hydrocarbons, leading to increased false positive rates (so-called ‘cheater’ populations). Overcoming this requires either a) spatial separation of variants, vastly reducing potential throughput; b) rapid detection of improved variants before significant product leakage occurs or c) the use of counterselection methods to identify and remove cheating cell populations.

The use of biosensor-based approaches that are not coupled to growth relies on the availability of specialist equipment to perform high-throughput screening, such as FACS for single-cell quantification of fluorescence and sorting. If specialist equipment is not available, screening throughput has to be lowered several orders of magnitude to allow manual microtiter or agar plate screening. Increasing the throughput of screening methods therefore also increases procedure complexity and costs, lowering accessibility. Since detrimental mutations are far more common than desirable ones, enzyme screening campaigns inevitably result in the observation of mostly low-performing isolates. Whilst this could be valuable for gathering information about the enzyme’s sequence-structure–function relationships, especially to generate labelled data for AI-based rational mutagenesis approaches, it still means that most resources are spent on observing non- or poorly functioning variants.

## Growth-coupled selection methods

Another DE strategy involves coupling the activity of the target enzyme to cell fitness. Host cells expressing enzyme variants with increased activity gain a growth advantage and outcompete their neighbours, mimicking the principles of natural selection to enrich high-performing variants within a diverse population. Selection methods are often based on the target enzyme activity rescuing a natural or engineered metabolic deficiency. When growth is dependent on the activity of an enzyme the enzyme is deemed to be growth-coupled (for a comprehensive overview of growth coupling principles (see [2]). Growth-coupled approaches offer a low-cost and procedurally simple method for enzyme engineering, as they consolidate both screening and selection steps into a single process operation. As growth (rate) is the output metric, populations of variants can be enriched for those conferring growth benefits using selective serial passages of the population, or by selective cultivation in turbidostats. The throughput of such approaches is usually only limited by the transformation efficiency of the host, which can be in the range of 10^8^–10^10^ CFU/μg DNA for *E. coli* [64]*,* or by the target’s mutation rate in the case of in vivo mutagenesis [34]. Since all successfully transformed hosts are simultaneously assessed for their fitness in selective conditions, the throughput of growth-coupled selection is amongst the highest of all existing enzyme engineering approaches, whilst only requiring minimal resources. One caveat to this is that the success rate of identifying a beneficial variant from a pool of competing variants can be limited by clonal interference [23].

Directed evolution by selection is only as good as the imposed selection pressure and its stringency [5]. It is therefore imperative that the selection pressure for such an approach is chosen carefully and extensively characterised. When growth is coupled to product synthesis, it is important to establish the relationship between output growth (rate) and the target product biosynthesis rate to establish a dynamic range of the selection platform. This information will be useful in avoiding either a maximisation of growth rate with unevolved enzyme activity, leaving no selective pressure for further improvement of the enzyme, or a situation where enzyme activity is too low to rescue growth to begin with. To avoid the first issue, concentrations of the growth-coupled enzyme can be reduced by swapping constitutive promoters to lower-strength variants, reducing induction levels of inducible promoters, or by its fusion to a protein degradation tag. However, in most cases growth-coupled schemes are bottlenecked by the initial step of coupling growth solely to enzyme activity.

Hydrocarbon-producing enzymes vary in their activity, but most have catalytic activity far below those of strongly growth-coupled central metabolic enzymes [7]. For example, ADO is infamous for its sluggish activity, displaying turnover numbers of ~ 1 min^−1^ in vitro [57]. The light-driven fatty acid photodecarboxylase *cv*FAP was shown to be more efficient but still below the median turnover number of central and secondary metabolic enzymes, achieving a k_cat_ of ~ 0.9 s^−1^ [83]. Ensuring these relatively inefficient enzymes can rescue growth therefore requires their coupling to sensitive growth-rescue mechanisms, such as essential but low-flux metabolic pathways. Growth can also be made co-dependent instead of fully reliant on target enzyme activity, by supplying small amounts of the target product to help first establish growth. As the enzyme evolves towards higher activity this supplementation can be reduced until cell growth is fully dependent on the target activity. This approach of ‘weaning’ the cell off a rescue carbon source requires a comprehensive characterisation of the relevant dynamic range.

Hydrocarbons are a challenging class of molecules to target for DE approaches. Engineering cells to be dependent on hydrocarbon-producing enzymes therefore requires creatively designed selection methods, examples of which we have compiled below.

### Transcription factor-based growth rescue

**Fig. 3:**
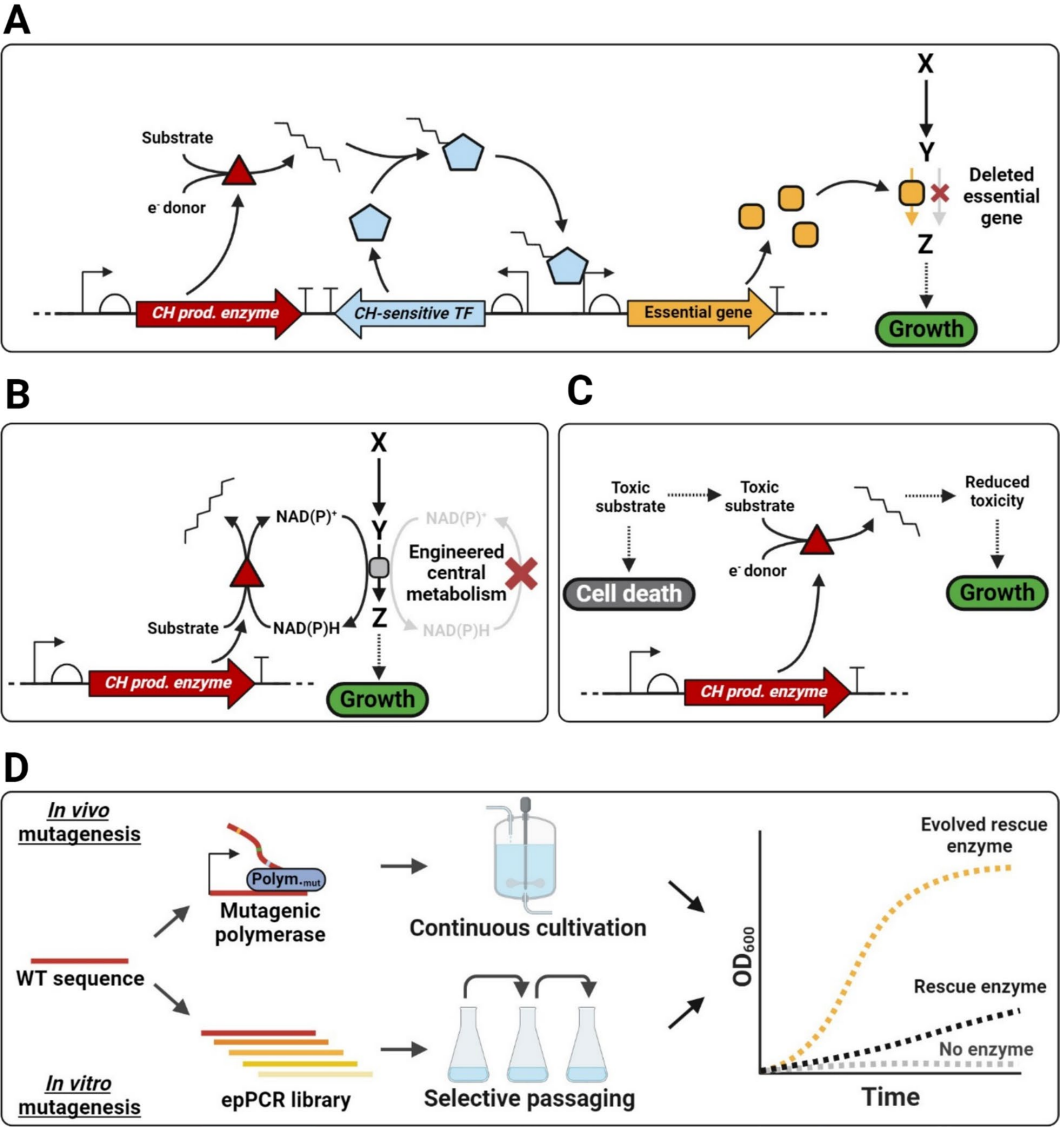
Selection methods for hydrocarbon-producing enzymes. **A** Schematic representation of a synthetic genetic construct consisting of a hydrocarbon production gene (CH prod. enzyme), hydrocarbon-detecting transcription factor (CH-sensitive TF) and a TF-responsive element. The responsive element consists of an essential gene to enable hydrocarbon-dependent essential gene expression. **B** Schematic of the principle of cofactor-based growth coupling. The microbial selection platform can be engineered to exhibit a deficiency in the oxidation or reduction of redox cofactors. These microbes are unable to grow unless a rescue reaction is introduced restoring redox cofactor cycling, in this example through the NAD(P)H-dependent reduction of substrate to hydrocarbon. **C** A toxic concentration of substrate can be used arrest growth or induce cell death unless the substrate is converted to the target hydrocarbon compound, resulting in growth and selection for enzyme variants with higher activity. **D** Evolution using these systems can be performed in vivo using continuous mutagenesis tools and continuous cultivation (top). Alternatively, more traditional methods such as error-prone PCR (epPCR) can be used with selection by serial passaging (bottom). Target enzyme activity is directly coupled to growth, so evolved enzymes confer a higher growth rate to the selection strain

Hydrocarbon-sensitive transcription factors (TFs) have been widely used in the context of enzyme screening, as described earlier. These hydrocarbon-sensitive TFs can be viewed as modular hydrocarbon detection units. They do not necessarily have to be linked to expression of a reporter molecule, such as GFP, but can instead be employed to drive expression of genes essential for growth. This way, the functionality of these TF systems can be transformed from that of a screening method to a selection method (Fig. 3A).

These growth-essential enzymes may either be of direct metabolic or structural relevance, involved in processing of important central metabolites or structural components (e.g. TF-based restoration of an enzyme producing cell wall components), or they may rescue growth by alleviating an external stressor, for example by detoxifying some molecule added to the growth media. The gene circuit architecture is common to both approaches, but the rescue mechanism is different. Notably, the latter approach allows for easily adjustable stringency of selection by varying the concentration of the toxic additive. This was exemplified in a prior study detailing the use of TF-based detection of small molecules to establish a growth-coupled system [25]. The authors coupled a TF sensitive to 1-butanol to the expression of tetracycline transporter gene *tetA*. The resulting strain could grow in tetracycline-containing medium only when supplemented with 1-butanol, or when 1-butanol was biosynthesised in vivo. This system was then used to engineer the RBS sequences of a mixed alcohol biosynthesis pathway. Although this work did not target aliphatic hydrocarbon synthesis, the underlying principle of the work demonstrates its viability.

Another approach requiring more complex gene circuitry was used to achieve a similar system with tuneable stringency. A convergently oriented inducible promoter system was constructed to modulate expression of a TF-regulated rescue gene by direct transcriptional interference, where the rescue gene was responsible for synthesis of cell wall components [42]. This system was used to select for alkane/alkene biosynthesis in *Acinetobacter baylyi*, using its native alkane-sensitive alkR/P_alkM_ transcriptional regulator and promoter system [42]. The *A. baylyi* strain was first made auxotrophic for the essential amino acid lysine by knocking out 4-hydroxy-tetrahydropicolinate reductase (*dapB*). This gene was then re-integrated into the chromosome but under control of the alkane-sensitive alkR/P_alkM_ system, in place of the native alkane monooxygenase *alkM*, which was removed to prevent degradation of hydrocarbon product.

Growth of this strain could be rescued either by direct supplementation of lysine in the medium, or by addition of mineral oil, inducing expression of the essential gene and thereby complementing growth. Using this system, a mixed population of *A. baylyi* strains transformed with low-, medium-, and high-producing alkane biosynthesis plasmids were shown to be enriched for the high alkane production plasmids over time. When assessed separately, strains with high-producing alkane biosynthesis plasmids displayed increased growth rates and decreased lag phases compared to those transformed with low-producing alkane production plasmids.

This study demonstrated that TF-based hydrocarbon detection could also be used to establish a selection system for hydrocarbon production, as opposed to just a screening method. Due to the growth-based selection principle only a selective cultivation of the strain would be required to achieve population enrichment of positive clones, considerably simplifying the process compared to a screening method. This approach does still suffer from some of the same limitations as TF-based screening approaches, including potential cross-feeding of product in the population and the dependence on TF specificity and genetic circuit integrity.

### Cofactor-based growth coupling

A relatively new concept in the field of growth-coupled enzyme engineering is to use redox cofactor auxotrophy as a selection method [62]. In this approach, strains unable to grow due to an imposed deficiency in the reduction or oxidation of a certain cofactor pair (e.g. NAD^+^/NADH or NADP^+^/NADPH) can be used to select for enzymes that alleviate this redox imbalance. This method offers a way to couple growth to redox cofactor-requiring enzymes or pathways, without having to use the target product as the selective molecule directly (Fig. 3B). Since redox cofactors are the selected molecules in this approach, issues caused by low solubility and poor compartmentalisation of hydrocarbon products can be avoided.

Aliphatic hydrocarbons can be biosynthesised through carbon chain-elongating steps in metabolism, such as fatty acid biosynthesis or reverse beta oxidation. These pathways involve several reductive reactions requiring significant input of NAD(P)H, making them viable targets for cofactor-based growth-coupling using strains deficient in the cycling of NAD(P)H back to their oxidised counterparts NADP^+^ or NAD^+^. Several hydrocarbon biosynthesis enzymes also require cofactors, such as AAR (NADPH, directly), ADO (NAD(P)H, ferredoxin-mediated) and OleT_JE_ (NAD(P)H, cytochrome P450 reductase). Redox cofactor auxotrophs have already been used to engineer a 3-hydroxy-acyl-CoA reductase [55], carboxylic acid reductase [44], P450 cytochrome [58] and a nitroreductase [79], to name several. The breadth of enzyme activity targetable by this DE approach makes it a promising technology for engineering hydrocarbon-producing enzymes.

Although this approach has not been used to engineer hydrocarbon production so far, we expect cofactor-based growth coupling to become a more widely used tool in the DE repertoire due to its unique product-independent property and improved resilience against inactivating mutations in the growth-coupling gene circuit.

### Substrate toxicity alleviation

Instead of selecting for the biosynthesis of a target product, the consumption of a substrate can also be linked to cell fitness if the substrate is toxic and the product less so. Consumption of the toxic substrate by the target enzyme alleviates its toxic effects, either reducing the growth lag phase of the host cell, improving its growth rate, or a combination of the two (Fig. 3C).

Although this approach has a limited scope, since the target enzyme must have a toxic substrate and a less toxic product, it provides a direct way to link enzyme activity to growth, making the gene circuit less prone to inactivation by the host. One study has successfully employed this approach to engineer aldehyde deformylating oxygenase (ADO). The aldehyde substrate for ADO is toxic to *E. coli* depending on concentration and carbon chain length. The authors exploited this property by adding inhibitory concentrations of aldehydes to *E. coli* cultures, where they found that ADO-expressing cells exhibited improved growth [53]. After a round of random mutagenesis, the authors identified an *E. coli* protease degradation target on the C-terminus of the protein, which when removed or protected by a His-tag significantly improved the concentration of soluble ADO protein in vivo. The authors used an engineered strain deficient in several native aldehyde reductases to ensure aldehyde consumption by the host was limited. This approach lends itself well to enzymes that catalyse the conversion of toxic substrates that are poorly, or not at all, degraded to non-toxic products by the host.

A second relevant study, although not focussing on aliphatic hydrocarbon production, demonstrated that the detoxification of medium-chain fatty acids can be used as a selective principle [36]. In this work, the authors engineered carboxylic acid reductases for improved activity against medium-chain fatty acids (MCFA), to generate the concomitant alcohols. One aspect of the work echoes the previous ADO-targeting study exploiting aldehyde toxicity, namely that endogenous pathways capable of detoxifying the substrate were removed. For example, deletions of a MCFA exporter and an aldehyde dehydrogenase were performed to ensure accumulation of alcohol product. Considering several hydrocarbon-forming enzymes use fatty acids directly as a substrate, this approach warrants further exploration.

For many hydrocarbon-producing enzymes, their substrate specificity in terms of carbon chain length is a major focal point for enzyme engineering. Substrate toxicity alleviation provides an attractive method to customise selective pressure towards specificity for a certain carbon chain length.

### Advantages and limitations of hydrocarbon selection methods

The use of selection methods for engineering hydrocarbon enzymes offers a way to perform ultra-high throughput DE with minimal requirements for complex equipment and delicate, often robotics-assisted, procedures. These methods are therefore highly attractive for applying selection to large combinatorial variant libraries, performing continuous mutagenesis, and evolving whole-cell systems towards improved product synthesis.

While the advantages are considerable, growth-coupled selection methods are challenging to establish. Some level of metabolic engineering is almost certainly required, and extensive characterisation of the resulting metabolic deficiencies must be performed before a DE campaign can be initiated. In addition, the catalytic rate of hydrocarbon biosynthesis enzymes is lower on average than essential enzymes in central metabolism [7]. Establishing growth-coupling solely to target enzyme activity therefore requires careful planning regarding which essential metabolic node to target, since this defines the dynamic range within which the selective pressure will operate. Establishing selection based on essential gene expression may therefore be a useful alternative to the direct metabolic coupling of enzyme activity, since expression strength can also be modulated through other means such as RBS swapping or using multi-copy constructs.

Besides establishing a selection method, ensuring the required selective pressures remain in place during DE procedures can be challenging as growth is often severely inhibited. Fundamentally, even with the most sophisticated growth-coupling methods, the researcher’s pursuit of “productivity” is imperfectly aligned with the host microbe’s pursuit of “survival”, the latter being achievable either by developing along prescribed evolutionary routes toward hydrocarbon production or by simply circumventing the genetic circuits put in place to encourage such evolution. Unlike lower-throughput DE methods leveraging external screening and selection machinery, growth-coupling DE methods rely more on constructed genetic circuits which are open to manipulation by the host organism. Preserving regulatory gene modules governing rescue gene expression against mutation, especially in the face of stringent selection pressure over many generations, is a major obstacle to development of this DE approach. Switchable expression by a hydrocarbon-sensitive TF can easily mutate to become constitutive, restoring rescue genes and returning the host to a high-growth phenotype independent of hydrocarbon productivity. Escape mutants disrupting the DE process will assuredly appear, requiring either an extremely stringent selection method incorporating contingencies for repair of compromised genetic circuits, or a counterselection method to combat cheating populations [27].

## Future perspectives

Directed evolution methods for hydrocarbon-producing enzymes are challenging to establish, often requiring preliminary genetic and protein engineering to establish high-throughput screening and selection procedures. Much ground has been covered in recent years, but several research areas remain unapproached.

The sensitivity, specificity and robustness of hydrocarbon biosensors has steadily improved over the past decade, with recent work demonstrating its potential for engineering the AAR-ADO pathway [18]. Despite these improvements, the majority of studies still apply them solely for fluorescent reporter gene expression, even though this approach requires specialised equipment for repeat rounds of external screening and sorting, precluding its use for continuous evolution. We envision these biosensors being used in growth-coupled designs, where the detection of hydrocarbons is coupled to the expression of gene (circuits) linked to growth. Although this would require more extensive initial strain engineering and characterisation, these growth-coupled selection methods can interrogate a vastly larger solution space of variants, while also offering reduced operational complexity and equipment requirements, potentially accelerating the discovery of improved enzyme/pathway variants.

The use of substrate toxicity as a selection method for aliphatic hydrocarbon production has only been used to engineer ADO [53], but other potential targets for this approach are fatty acid decarboxylating enzymes such as OleT_JE_, UndA, UndB and *cv*FAP. Short- and medium-chain fatty acids are toxic to *E. coli* and *S. cerevisiae* above certain concentrations depending on their chain length [36, 73], meaning that enzymes catalysing their conversion to less toxic alkanes or alkenes could potentially be engineered using this approach. Efficient production of gaseous hydrocarbons such as propane and butane is also highly sought after, but performing DE using the gasses themselves as the target enzyme product is challenging due to a lack of compartmentalisation (assuming that their intracellular concentration determines the DE system’s response). Alleviating short-chain fatty acid toxicity as the selective principle may enable easier engineering of these enzymes. When using *E. coli* as a host for enzyme selection, exogenous fatty acids can also be easily excluded from cell metabolism by deletion of *fadD*, avoiding their activation to acyl-CoA and subsequent degradation.

Studies focussed on engineering hydrocarbon biosynthesis have mostly reported either host-level metabolic engineering (e.g. carbon flux improvements, redox balancing) or rational mutagenesis and screening specifically targeting only terminal hydrocarbon synthesis enzymes. However, when hydrocarbons are synthesised from a sole carbon source (i.e. the substrate of the terminal enzyme is not supplemented), some of the DE methods set out in this review could be used to evolve entire metabolic pathways and other cellular processes towards improved hydrocarbon synthesis. Besides improving bioprocess performance using common substrates such as glucose, growth-coupled designs enable the simultaneous engineering of product biosynthesis and substrate catabolism. This can be exploited to evolve cell factories towards optimal assimilation and conversion of CO_2_-derived carbon sources. In vivo continuous targeted hypermutation [60] or whole-genome mutagenesis tools [91] represent suitable technologies for performing this type of evolutionary engineering towards improved product synthesis, though exempting from mutation the crucial gene circuitry responsible for growth-coupling remains an obstacle. Consequently, in vivo mutagenesis coupled with a high-throughput DE approach has not been performed in the context of hydrocarbon biosynthesis.

This review focussed on novel methods to engineer (terminal) enzymes involved in hydrocarbon biosynthesis, which we consider a crucial bottleneck for maturation of the field. Indeed, full exploitation of the screening and selection methods described in this review may offer considerable benefits over currently used strategies. It is acknowledged, however, that in the context of hydrocarbon biosynthesis, there may be physiochemical limitations hindering the success of enzyme engineering campaigns. For example, the often poor solubilities exhibited by substrates and products of hydrocarbon biosynthetic pathways may fundamentally limit catalytic performance, at least when the enzyme(s) of choice is/are utilised in vivo. Strategies including the use of cell-free systems with optimised media for hydrocarbon biosynthesis could remove some of these limitations. Alternatively, bespoke reactor design could ensure the reaction environment is better suited for hydrocarbon biosynthesis through the use of pressurisation or biphasic production processes. These and other strategies could help ensure that the enzyme fitness landscape being traversed during directed evolution remains free of obstacles.

## Conclusion

Living systems are undoubtedly the most sophisticated manufacturing platforms on earth; able to adapt, self-repair, and transform diverse substrates into complex structures under mild conditions with unmatched molecular precision. But the sheer physiological complexity of living systems means biological engineers are typically operating with incomplete knowledge. An engineering approach able to rapidly explore a broad solution space, while avoiding the need for extensive knowledge of all factors at play, represents a powerful tool for optimising such a system. Directed evolution is such a tool; a massively parallel black-box approach to the traditional engineering design-build-test-learn cycle, attempting to emulate and expedite a natural strategy that can be observed in our living world.

Biosynthesis of drop-in compatible hydrocarbon fuels is a promising field, but has struggled to establish processes with high titres, rates, and yields. Even though several hydrocarbon-producing enzymes and their related pathways have been identified and extensively characterised over the past decade and a half, the limited activities of these enzymes make them unsuited to commercial use. A considerable bottleneck to the improvement of hydrocarbon-producing enzymes and, more holistically, industrially useful hydrocarbon-producing strains, is a lack of appropriate screening or selection methods to identify improved variants in a high-throughput manner. While directed evolution seems well-suited to tackle these problems, designing directed evolution strategies for hydrocarbon synthesis is challenging and requires creative implementation of multiple facets of synthetic biology. In this review, we have summarised the development of directed evolution methods for hydrocarbon production and discussed their advantages and limitations.

The most widely reported directed evolution systems utilise a screening method based on a hydrocarbon-sensitive genetic construct driving expression of a fluorescent reporter protein. Although these systems initially displayed low selectivity and sensitivity, considerable improvements have been made over the years. These efforts have resulted in screening systems that can now detect alkanes with broader chain length specificity, while also exhibiting improved dynamic range and lower leakiness. As a result, recent studies have reported the application of these tools for the directed evolution of ADO, achieving promising improvements in activity.

A relatively unexplored method for engineering hydrocarbon production is directed evolution by selection. Many of the limitations of screening approaches can be circumvented using selection methods, but these may require more extensive genetic engineering and characterisation to establish. The selection methods summarised in this work include selection by substrate toxicity alleviation, cofactor-based growth coupling, and hydrocarbon-sensitive transcription factors driving expression of essential genes. Besides their use as enzyme engineering platforms, strains displaying a growth dependence on hydrocarbon biosynthesis may be valuable assets in the context of bioprocess stability and antibiotic-free biomanufacturing. Despite these attractive features, the body of work encompassing growth-based selection for hydrocarbon biosynthesis is minimal and remains an area with high potential.

In summary, the field of drop-in hydrocarbon biosynthesis is steadily moving from discovery and characterisation of useful genetic parts, through to metabolic and protein engineering efforts to improve the useful function of said parts, towards leveraging the principles of directed evolution to accelerate the traditional engineering design-build-test-learn cycle. By fully utilising the continuously expanding toolbox of synthetic biology, screening and selection of hydrocarbon-producing enzymes and organisms will be made increasingly accessible. Hopefully, this will accelerate the development of novel enzyme variants and new, industrially relevant microbial strains that can help drive sustainable hydrocarbon synthesis towards commercial viability.

## Data Availability

No datasets were generated or analysed during the current study.
